# Viral burden and diversity in acute respiratory tract infections in hospitalized children in wet and dry zones of Sri Lanka

**DOI:** 10.1371/journal.pone.0259443

**Published:** 2021-12-17

**Authors:** J. A. A. S. Jayaweera, A. J. Morel, A. M. S. B. Abeykoon, F. N. N. Pitchai, H. S. Kothalawela, J. S. M. Peiris, F. Noordeen

**Affiliations:** 1 Department of Microbiology, Faculty of Medicine and Allied Sciences, Rajarata University of Sri Lanka, Saliyapura, Sri Lanka; 2 Department of Microbiology, Faculty of Medicine, University of Peradeniya, Peradeniya, Sri Lanka; 3 Teaching Hospital, Gampola, Gampola, Sri Lanka; 4 School of Public Health, University of Hong Kong, Pok Fu Lam, Hong Kong; Health Directorate, LUXEMBOURG

## Abstract

The present study was done to identify the viral diversity, seasonality and burden associated with childhood acute respiratory tract infection (ARTI) in Sri Lanka. Nasopharyngeal aspirates (NPA) of hospitalized children (1 month—5 years) with ARTI were collected in 2 centers (wet and dry zones) from March 2013 to August 2014. Respiratory viral antigen detection by immunofluorescence assay (IFA) was used to identify the infecting viruses. IFA negative 100 NPA samples were tested for human metapeumovirus (hMPV), human bocavirus and corona viruses by polymerase chain reaction. Of the 443 and 418 NPAs, 37.2% and 39.4% were positive for any of the 8 different respiratory viruses tested from two centers studied. Viral co-infection was detected with respiratory syncytial virus (RSV) in both centers. Peak viral detection was noted in the wet zone from May-July 2013 and 2014 and in the dry zone from December-January 2014 suggesting a local seasonality for viral ARTI. RSV showed a clear seasonality with a direct correlation of monthly RSV infections with rainy days in the wet zone and an inverse correlation with temperature in both centers. The case fatality rate was 2.7% for RSV associated ARTI. The overall disability adjusted life years was 335.9 and for RSV associated ARTI it was 241.8. RSV was the commonly detected respiratory virus with an annual seasonality and distribution in rainy seasons in the dry and wet zones of Sri Lanka. Identifying the virus and seasonality will contribute to employ preventive measures and reduce the empirical use of antibiotics in resource limited settings.

## Introduction

Acute respiratory tract infection (ARTI) represents one of the most common acute illnesses in childhood. ARTIs range from common cold, a mild, self-limited catarrhal syndrome to life threatening lower respiratory tract infections [1]. Viruses account for most ARTIs and several viruses are associated with ARTI [2]. The most frequently reported respiratory viruses in newborns and children under 5 years are respiratory syncytial virus (RSV), parainfluenza virus types 1, 2, 3,4 (PIV-1, -2, -3, and 4), adenovirus (AdV), influenza virus types A (Inf-A), B (Inf-B), human corona virus (hCoV), enteroviruses, human boca virus (hBoV), and human metapeumovirus (hMPV) [3, 4].

Viruses cause most of the ARTI but a small percentage of these infections results in severe or fatal disease. Viral ARTI lead to secondary bacterial infections in healthy children by lowering the immunity in the respiratory tract allowing the invasion of bacteria [5, 6]. Most respiratory viruses cause lower respiratory tract infection (LRTI) of different severity with a wide range of respiratory manifestations including severe pneumonia and bronchiolitis. However, detection of viral etiology of ARTI will be useful since it contributes to minimize the use of antibiotics, which is unwarranted in viral ARTI in the absence of bacterial co-infections. Prematurity, immuno-compromised state and congenital heart or lung diseases are the risk factors for severe LRTI in children. RSV infection in children with high risk often leads to severe LRTI requiring stringent monitoring to reduce morbidity and mortality [7–9].

The ARTI associated disease burden is greater than that of any other infections [10]. In 2014, 18%, 15%, and 11% of the mortality in children younger than 5 years of age was caused by ARTIs, diarrhea and malaria, respectively [11]. ARTIs account increased mortality and morbidity in children imposing a burden on the health care systems [12–14]. Prevention of viral ARTI is important as antivirals and vaccines are not available against many respiratory viruses [7].

Hand hygiene, respiratory etiquettes, use of personnel protective equipment are effective in preventing the spread of respiratory viral infections. In addition, contact precautions play a crucial role in preventing RSV and PIV infections in healthcare settings; droplet precautions are useful in preventing Inf-V and rhino viral infections; contact and droplet precautions are useful in preventing adenoviral respiratory infections. Early viral diagnosis and active surveillance are essential for the identification of infected cases and symptomatic management when necessary [12–14].

The viral detection in ARTI depends on many factors, such as sample processing and testing, disease severity, diagnostic methods, and the seasonal trends with the local climate [15]. There is a seasonal variation in viral incidence, which is higher during the colder months in countries with temperate climates [16]. In countries with tropical climates, the seasonality varies according to the temperature-dependent local weather pattern such as humidity, and / or rainfall [17]. Global warming carries a profound change in earth’s climate and major changes in the atmosphere will make an impact on the biosphere, and the environment [18]. Conversely, climatic variations and extreme weather events also cause profound impact on the incidence of viral ARTI in a given area or a region. Detailed epidemiological studies on the viral etiology of ARTI have not been performed in children in different climatic zones of Sri Lanka. Thus the current study was undertaken to determine the viral etiology of ARTI and associated disease burden in childhood ARTI in two centers from dry and wet zones of Sri Lanka.

## Materials and methods

### Patients and clinical specimens

**Fig 1:**
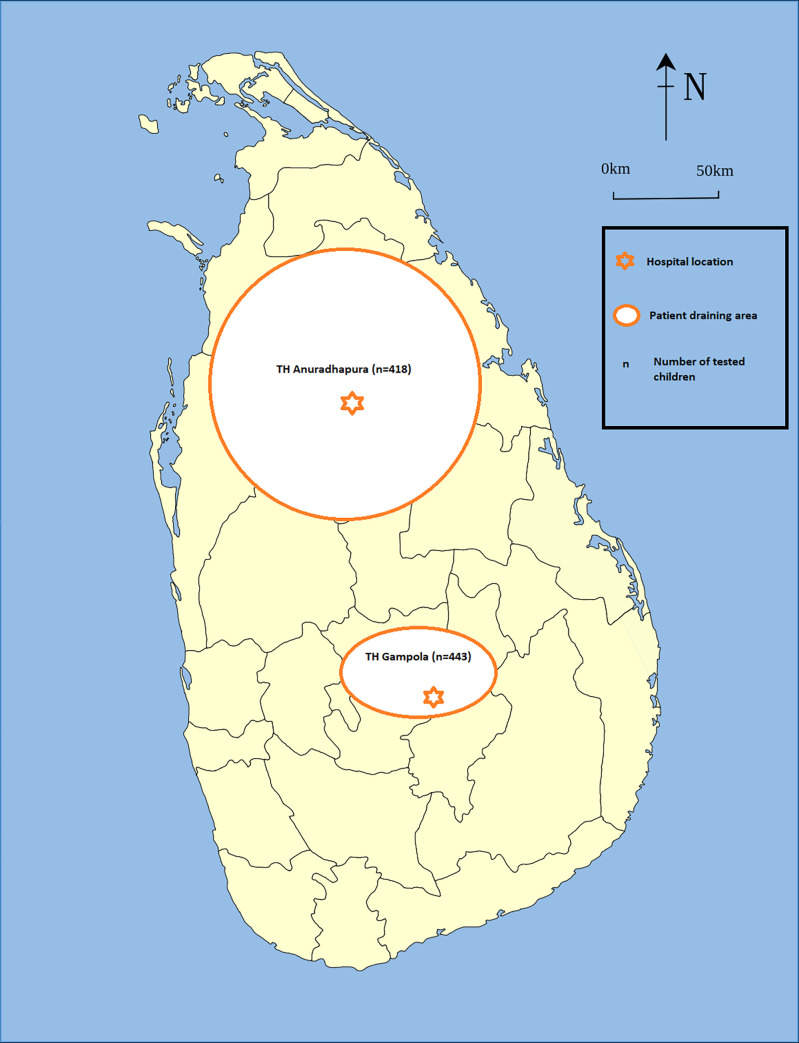
A map of Sri Lanka with sampling sites (THA and THG), number of children recruited in each center (n) and patient draining area. THA–Teaching hospital, Anuradhapura; THG–Teaching hospital, Gampola.

Ethical approval (Permit No 2012/EC/24) for the study was obtained from Faculty of Medicine, University of Peradeniya, Sri Lanka. This was a prospective cross-sectional study in hospitalized children with ARTI from 1 month to 60 months. The study was performed at the children’s ward of the Professorial Unit of the Teaching Hospital, Anuradhapura (THA) located in the North Central Province (Dry zone), and the Teaching Hospital, Gampola (THG) located in the Central Province (Wet zone) of Sri Lanka (Fig 1).

To study the year-round incidence of different viral ARTI, recruitment of participants for the study was done on consecutive days throughout the year for 18 months from March 2013 to August 2014. Hospitalized children were recruited with ARTI with severe acute respiratory illness (SARI) using the case definition of the World Health Organization (WHO) [19]. The clinical diagnosis of ARTI was done by the consultant pediatricians using the ICD-10 2012 guidelines [20]. A child was included in the study after obtaining the guardian’s or parents’ consent following the objectives explained in English or the local languages. Patient’s history and data on clinical signs and symptoms, demography and risk factors for acquiring ARTI were collected using an investigator administered questionnaire by interviewing the parent or guardian with the informed written consent. Then the nasopharyngeal aspirates (NPA) were taken using the recommended mucous aspirator with minimal discomfort to the child by the pediatrician or his trained medical staff to test for the infecting respiratory virus.

### Detection of respiratory viruses

NPA was collected with sufficient number of respiratory nasopharyngeal epithelial cells (NPC) as these cells have the virus infection. NPA was then released into 8 mL phosphate buffered saline (PBS) and transported on ice to the laboratory (4°C) within a few hours of collection. Indirect immunofluorescence assay (IFA) was performed using the D3 (USA) [21] with comparable sensitivity with culture and a required specificity as stated by the manufacturers. IFA covered screening for 8 respiratory viruses including RSV, AdV, PIV-1, PIV-2 and PIV-3, Inf-A and Inf-B, and hMPV and then the IFA screening positive NPA were subjected to identify the infecting virus using monoclonal antibodies provided by the D3 Ultra DFA Respiratory Virus Screening and ID Kit (USA). Stained slides were examined under UV-Epi fluorescence microscope (Leads, Germany) and intracellular nuclear and/or cytoplasmic apple green fluorescence emitting cells were recorded as virus infected. IFA negative samples were then vortexed, transferred into 1.5 mL Eppendorf tubes and stored at -70°C for polymerase chain reaction (PCR) to detect hMPV, hBoV, and coronaviruses (CoV) [22].

### PCR to detect hMPV, hBoV and CoV

To detect respiratory viral co-infections, a qualitative PCR was performed for hMPV, hBoV and CoVin100 RSV positive children. Moreover, 100 selected IFA negative children’s samples were subjected to PCR to detect hMPV [23], hBoV [24] and CoV [25] as primary etiological agents in ARTI using previously published protocols.

All the laboratory tests for the study were done in the Diagnostic and Research Virology Laboratory of the Department of Microbiology, Faculty of Medicine, University of Peradeniya, Sri Lanka.

### Statistical analysis

Data obtained was double entered into a spreadsheet in Microsoft^®^ Excel and cleaned for double or wrong entries. Associations between categorical and continuous variables were analyzed using the Chi square tests or Fisher’s exact tests, and the 2-tailed Student’s *t* test or Wilcoxon rank-sum test as appropriate.

Demographic data, clinical features, disease spectrum, mean hospital stay and use of antibiotics in RSV, PIV-1, -2 and -3, AdV, Inf-A, Inf-B, and hMPV associated ARTIs were expressed in measures of central tendency (Table 1 and Supplementary 1 in S1 File). Multivariable analyses were performed using a step wise logistic regression analysis to assess the risk factors for developing RSV associated ARTI vs other tested individual viral ARTIs. Initially, a univariate analysis was done and *p*<0.1 was selected for the multivariate analysis and modeling. Modeling was done for RSV positive vs. positive for any of other seven viruses. Variables such as age at hospitalization (nearest month), duration of the disease (days), gender, ethnicity (Sinhala, Tamil, Moor, and other), weight (kg), and Hb%, chronic malnutrition (height-for-age ≤ 2SD was taken while weight-for-age ≤2SD also considered to assess malnutrition), gestational age considering the maturity of the mother, mode of delivery (vaginal or lower segment cesarean section), presence of underlying medical conditions (congenital heart disease-CHD; chronic lung disease-CLD, asthma, cystic fibrosis) and genetic disorders: Down’s syndrome-trisomy 21; neuromuscular disorders and pre-existing respiratory tract morbidity, passive smoking (the involuntary inhalation of cigarette smoke from other smokers or the child’s father), having house hold pets, presence of indoor smoke through fire wood used for cooking / houses without chimney and outdoor air pollution through construction activities outdoor or industries), crowding (child’s living area <24 m^2^) [26], guardian’s/parent’s education (<grade 8, up to advanced level and graduates), experience as a caregiver (first child or experience in caring>1 child) and occupation. Odds ratio was calculated for these variables with consideration of significance at *p* value of <0.05. Multivariate odds ratios with 95% CIs that did not include 1.0 were considered as significant and the continuous variables were expressed as measures of central tendency. Children with viral co-infections were excluded in clinical data analysis.

**Table 1:** Characteristics of hospitalized children with ARTI from THA and THG study samples with respect to 8 major respiratory viruses.

Viral characteristics	RSV		PIV-1		PIV-2		PIV-3		AdV		Inf-A		Inf-B		hMPV		*P*
Number of children studied	(n = 94)	(n = 85)	(n = 6)	(n = 11)	(n = 12)	(n = 16)	(n = 8)	(n = 8)	(n = 17)	(n = 17)	(n = 8)	(n = 11)	(n = 5)	(n = 8)	(n = 15)	(n = 9)	
Hospital recruited	THG	THA	THG	THA	THG	THA	THG	THA	THG	THA	THG	THA	THG	THA	THG	THA	
Number of children with co-infections	13	13	3	3	3	4	0	0	4	4	1	1	0	1	4	4	0.01
**Demographic characteristics**																	
Age at presentation, mean months ± SD	10±9.1	11±9.2	12±8.1	13±8.9	14±9.1	13±8.5	12±6.1	13±7.1	23±12.3	24±12.9	22±9.4	23±8.7	14.1±5.1	13±7.1	24.2±13.1	23±10.3	0.01
Male: female	60:34	53:32	3:3	6:5	7:5	10:6	5:3	5:3	10:7	4:3	5:3	7:4	3:2	5:3	9:6	5:4	0.03
**Clinical characteristics**																	
Fever	51	45	5	9	10	14	7	7	9	10	8	10	4	7	14	8	0.02
Highest body temp ≥ 39°C	34	32	4	5	4	6	4	6	9	8	6	4	3	4	7	8	
Duration of illness ≥7 days	35	35	3	4	5	7	4	5	5	6	4	4	3	2	5	7	>0.05
Tacypnoea	52	49	3	2	4	5	3	4	3	4	4	4	3	1	13	14	0.03
Chest recession	50	47	2	4	11	12	4	5	3	4	3	4	4	4	3	1	0.04
Rales	64	62	4	5	8	10	2	3	3	3	3	2	2	3	3	4	0.03
Wheezing	37	33	3	4	5	8	4	5	5	6	4	4	3	2	5	7	>0.05
Diarrhea	2	0	0	0	1	0	0	0	4	9	0	0	0	0	0	0	0.03
Conjunctivitis	0	0	1	0	1	0	1	0	6	9	0	0	1	1	0	0	0.04
**Clinical diagnosis**																	
Upper ARTI																	
Common cold	12	14	1	1	3	4	0	1	2	3	1	2	2	1	1	1	0.04
Acute sinusitis	1	1	0	1	1	0	1	1	0	0	0	0	1	1	0	0	>0.05
Acute pharyngitis	0	1	0	0	1	1	1	1	2	2	1	1	0	0	0	0	>0.05
Acute tonsillitis	1	0	0	0	0	1	0	0	0	1	0	0	0	1	0	0	>0.05
Acute laryngotracheitis	2	1	0	0	0	0	0	0	1	1	1	0	0	0	0	0	>0.05
Un specified upper ARTI	1	1	0	1	1	1	1	1	1	2	0	1	0	0	0	0	>0.05
**Lower ARTI**																	
Acute bronchiolitis	46	42	1	2	2	4	2	1	2	4	2	3	1	1	7	2	0.02
Mild	12	11	1	0	0	1	1	0	1	2	1	1	0	0	2	1	0.03
Moderate	15	11	0	1	1	2	1	0	1	2	1	0	1	0	2	1	0.04
Severe	19	20	0	1	1	1	0	1	0	0	0	2	0	1	3	0	0.03
Acute Bronchitis	3	2	0	0	0	0	0	0	0	0	1	0	0	0	0	0	>0.05
Pneumonia Lobar	3	3	1	1	0	2	1	0	1	1	1	1	0	1	1	1	>0.05
Broncho	3	3	1	1	1	1	1	1	1	1	1	1	0	0	1	1	>0.05
Un specified lower ARTI	2	1	0	1	0	0	0	0	1	1	0	1	0	0	1	0	>0.05
Asthma exacerbation	3	3	0	0	0	1	0	1	1	1	0	1	0	1	1	0	>0.05
Total hospital stays days ± SD	4±4.1	4±3.8	3±2.1	3±2.6	3±2.1	3±2.2	3±2.3	3±2.4	4±2.1	4±2.3	3±2.1	3±2.1	4±2.1	4±2.3	5±3	5±2.8	>0.05

THG–Teaching hospital, Gampola; THA–Teaching Hospital Anuradhapura; ARTI–Acute respiratory tract infections; SD–Standard deviations; RSV–Respiratory syncytial virus; PIV– 1,2 &3; Parainfluenza virus 1,2 &3; Ad V–Adenovirus; Inf-A–Influenza A; Inf-B–Influenza B; hMPV–Human metapneumovirus. *P* value was calculated in multivariate analysis and P< 0.05 was considered significant.

A “peak” in virus activity was defined when the monthly proportion was ≥10% during the study period. The correlation between the monthly incidence of overall viral ARTI and climatic factors (mean atmospheric temperature in a given month (mTm in °C), mean relative humidity in a given month (mRH in %), and mean number of rainy days in a given month, windspeed and direction and atmospheric pressure) was determined using Spearman correlation coefficient and multiple linear correlation coefficient. Further a separate analysis was done between the monthly incidence of RSV associated ARTI, and climatic factors (Supplementary 4 in S1 File). In addition, the effects of climatic factors and their interaction were calculated using the geographical detector method. The geo-detector *q*-statistic measures the degree of special heterogeneity of a particular variable (Y) and the determinant power of an explanatory variable (X) of Y; the value of *q*-statistic is strictly within 0, 1 [27]. The *q*-statistic was calculated to evaluate the spatially stratified heterogeneity (Supplementary 5 in S1 File) as described in Xu et al. [28].

Disability adjusted life years (DALYs) was calculated using the Health Statistics and Information Systems WHO, 2000–2011 [29]. The statistical analysis was done using the Statistical Analysis System (SAS), Version 9.1 [30].

## Results and discussion

### Children with ARTI

The viral, demographic and clinical characteristics data of the study are summarized in Table 1 and Supplementary 1 in S1 File. A total of 418 and 443 children were included over the period of 18 months in THA and THG, respectively. Of the tested, 165 children were positive for respiratory viral antigen by IFA from each of the center for one or more viruses. In both study centers, the number of boys with ARTI was significantly higher compared to girls with ARTI (*p* = 0.03). Considering the ethnicity in THA and THG study samples collectively, the number of Sinhalese (ethnic majority in Sri Lanka) children with ARTI was significantly higher (*p* = 0.02, 0.01) compared to Tamil (an ethnic minority in Sri Lanka) and Muslim (another ethnic minority in Sri Lanka) children. Compared to THA, in the THG study sample, number of Tamil children with ARTI was significantly higher than other ethnic groups (*p* = 0.03). Considering the residential areas of THA and THG study samples, the number of children with ARTI from the rural areas (away from the urban and suburban limits) (*p* = 0.03) was higher than that from the semi-urban and urban areas, respectively. In the THG study sample, many children with ARTI were from semi-urban areas when compared to the THA study sample (*p* = 0.04).

### Children with viral ARTI

Viral ARTI was more common in 12–24 months-old children in both study centers compared to 1-≤12 and ≥ 24-≤ 60-month-olds. No significant differences were noted in the distribution of viral ARTI in different age categories between the two study centers.

Body weight was in median to -1 SD to -2 SD in THA and -1 SD in THG study samples. Blood hemoglobin (Hb)% was 9.1 ± 1.9 g/dl in the THA and it was 9.3±2.1 g/dl in the THG. Body weight and Hb% were not significantly different among children with ARTIs or viral ARTIs between the two study centers. All children have had the routine vaccine coverage from the national immunization program, which does not include immunization against influenza. Moreover, none of the study participants had vaccination against influenza.

### Clinical characteristics

Clinical characteristics of respiratory virus positive children are summarized in Table 1. Multiple viral infections were detected in 29 children. In both centers, co-infection with RSV was significantly higher compared to other viral co-infections (*p* = 0.03).

The most important symptoms in children for mothers to seek medical assistance were rapid respiratory rate (60%), fever (48%), wheezing (33%), and anorexia (20%). When compared to PIV-1, PIV-2, PIV-3, Inf-A, Inf-B, and hMPV infections, fever was less frequently observed in children with RSV or AdV infections (*p*<0.02). When compared to PIV-1, PIV-3, AdV, Inf-A, Inf-B, and hMPV infections, respiratory rales were frequently auscultated in children with RSV or PIV-2 infections (*p*<0.03). Chest recession was frequently observed in children with RSV or PIV-2 infection (*p*<0.03). Compared to RSV, PIV-1, PIV-2, PIV-3, Inf-A, Inf-B, and hMPV infections, conjunctivitis (*p*<0.04) and diarrhea (*p*<0.03) were frequently observed in children with AdV infection. Tachypnea was frequently observed in children with RSV or hMPV infections compared to PIV-1, PIV-2, PIV-3, AdV, Inf-A, and Inf-B infections (*p*<0.03). Bronchiolitis was frequently observed in children with RSV infections compared to PIV-1, PIV-2, PIV-3, AdV, Inf-A, Inf-B, and hMPV infections (Supplementary 2 in S1 File) (*p*<0.02). Children in all age category had bronchiolitis, however, bronchiolitis was frequently noted in children between 1 and 24 months (*p* = 0.03). There was no difference in the duration of illness in different types of viral ARTI. Past history of ARTI predisposed the children to a current viral infection (*p* = 0.04).

### Distribution of viral ARTI

**Fig 2:**
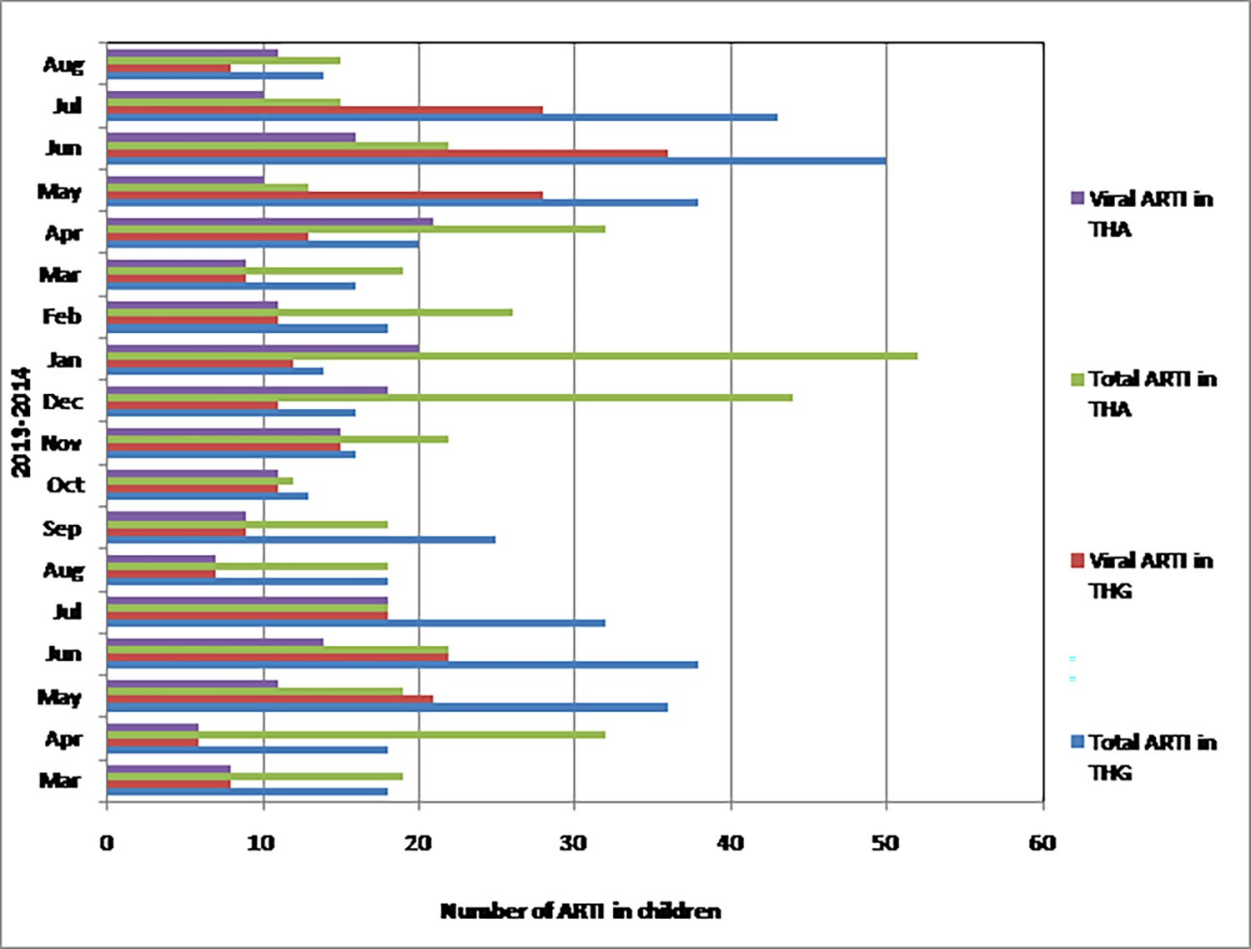
Monthly distribution of ARTIs in children less than 5 years in THA and THG from March 2013 to August 2014. ARTI–Acute respiratory tract infection; THA–Teaching hospital, Anuradhapura; THG–Teaching hospital, Gampola.

**Fig 3:**
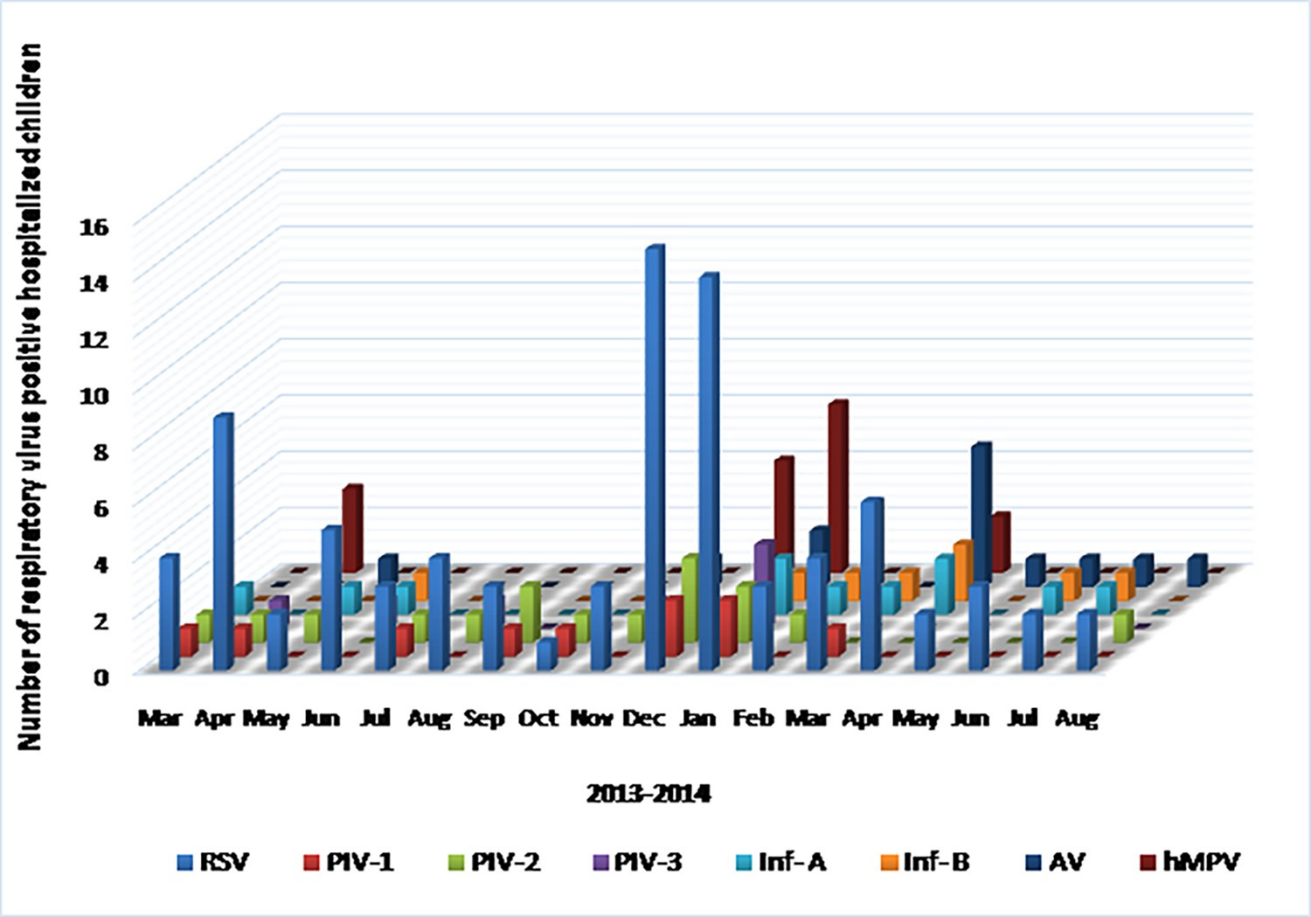
Pattern of distribution of viral ARTIs in children less than 5 years in THA from March 2013 to August 2014. ARTI–Acute respiratory tract infection; THA–Teaching hospital, Anuradhapura; RSV–Respiratory syncytial virus; PIV-1, 2 & 3 –Parainfluenza virus 1, 2 & 3; Ad V–Adenovirus; Inf-A–Influenza A; Inf-B–Influenza B; hMPV–Human metapneumovirus.

**Fig 4:**
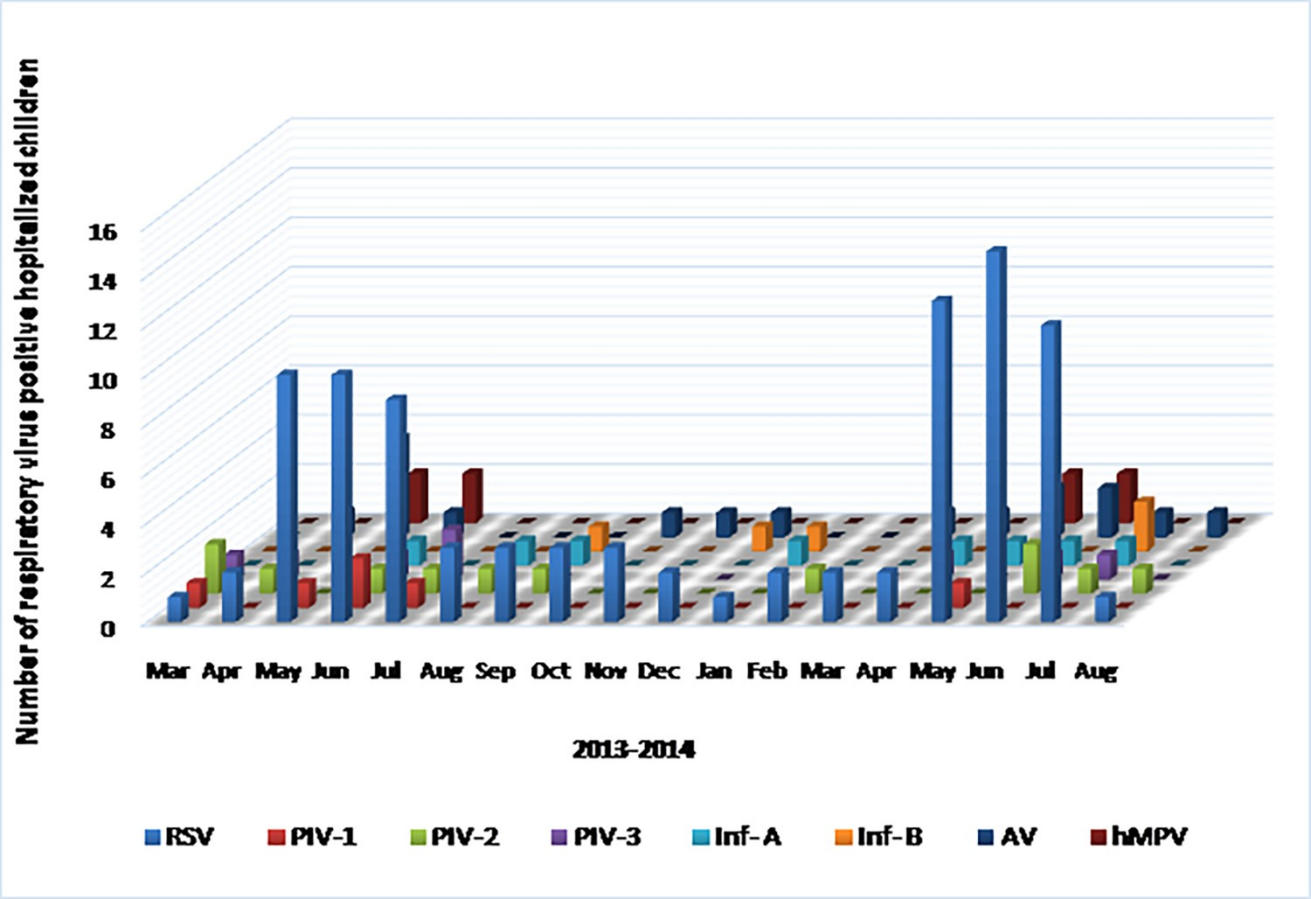
Pattern of distribution of viral ARTIs in children less than 5 years in THG from March 2013 to August 2014. ARTI–Acute respiratory tract infection; THG–Teaching hospital, Gampola; RSV–Respiratory syncytial virus; PIV-1, 2 & 3 –Parainfluenza virus 1, 2 & 3; Ad V–Adenovirus; Inf-A–Influenza A; Inf-B–Influenza B; hMPV–Human metapneumovirus.

Monthly distribution of ARTIs in THA and THG are shown in Fig 2. In both centers, ARTI was detected throughout the study period. ARTI peaked in the THA from December to January and in the THG from May to July. Overall, ARTI peaked during the rainy seasons. In the dry zone (THA), the peak ARTI was associated with north-east monsoon (from December to March) (Fig 3) and in the wet zone (THG), the peak ARTI was associated with south-west monsoon (from May to September) (Fig 4). In the dry zone (THA), two peaks of RSV infection were noted between December-January in 2013 (major peak) and in April in 2013 and 2014 (minor peak) (Fig 3). RSV infections were detected throughout the year in both centers. In the wet zone (THG), the peak of respiratory viral infections was noted from April to June in 2013 and 2014 (Fig 4).

In the THA, PIV-1 was detected between March and April in 2013 and December and January in 2014. In the THG, PIV-1infection was detected from April to June in 2013 and 2014. In the THA, PIV-2 was detected in March-June in 2013 and November-February in 2014. PIV-2 infection was detected from March to August in 2013 and May to August in 2014 in the THG. In the THA, PIV-3 was detected in June and August in 2013 and January in 2014. In the THG, PIV-3 infections were detected in April to June in 2013 and 2014.

AdV infection was detected in April, September to November in 2013 and February to August in 2014. In the THA, AdV was detected in May and December in 2013 and January to August in 2014 with a peak in April 2014. Inf-A infection was detected in May, July to August, and December in 2013 and March to June in 2014. In THA, Inf-A infection was detected in June in 2013 and January to April, June to July in 2014. Inf-B infections were detected in August, December in 2013 and June in 2014. In THA, Inf-B infections were detected in June 2013 and January to April, June to July in 2014.

In THA, hMPV infection was detected in March to April, November to December in 2013, and January to February in 2014. hMPV infection was detected in THG in March to April, November to December in 2013 and January to February in 2014. In both dry and wet zones, a similar seasonality was observed for hMPV infection. CoV and hBoV were not detected in tested RSV positive and 100 IFA negative samples from children clinically diagnosed with ARTI.

### Climatic factors and viral ARTI

Taken the explanatory power of the climatic factors for overall viral ARTI, the mean atmospheric temperature [0.41 (*p*<0.05)], the mean relative humidity [0.42 (*p*<0.05)] and the mean number of rainy days [0.46 (*p*<0.05)] had a greater influence. On the viral ARTI in THA and THG, the mean atmospheric temperature [0.39 (*p*<0.05), 0.44 (*p*<0.05)], the mean relative humidity [0.44 (*p*<0.05), 0.41 (*p*<0.05)] and the mean number of rainy days [0.46 (*p*<0.05), 0.48 (*p*<0.05)] had a greater influence. However, wind speed, wind direction and atmospheric pressure did not have an influence on viral ARTI.

Overall, the explanatory power of the climatic factors (atmospheric temperature, relative humidity and mean number of rainy days) was greater in boys than girls for each age group (1-≤12, 12–24 and ≥ 24-≤ 60 months). However, no significant difference was observed in the explanatory power of the climatic factors on viral ARTI between THA and THG (Supplementary 6 in S1 File).

Moreover, Spearman’s rank correlation and multiple linear regression showed a direct correlation of monthly viral ARTI cases with number of rainy days in both THA and THG centers and an inverse correlation with the mean atmospheric temperature. Additionally, regression analysis identified a further significant inverse correlation of viral ARTI cases with relative humidity in both centers. Monthly increase of one rainy day was associated with a 0.78 increase in monthly viral ARTI cases in THA and 0.88 increases in monthly viral ARTI cases in THG based on the multiple regression. However, in THA and THG, an increase of 1% of relative humidity and 1°C of temperature were associated with 1.91 and 1.83 and 2.82 and 2.76 decrease in viral ARTI cases, respectively. In THA, a total of 32.3% of explained variance (R^2^ = 0.323) in the number of monthly viral ARTI cases was attributed to number of rainy days in a month, relative humidity and temperature. In THG, a total of 33.3% of explained variance (R^2^ = 0.333) in the number of monthly viral ARTI cases was attributed to the climatic factors. Other climatic factors such as windspeed and wind direction and atmospheric pressure did not have an influence on viral ARTI.

### Climatic factors and RSV associated ARTI

Since RSV infection showed a clear seasonality with the highest number of cases, the association of RSV with climatic factors was further analyzed. For RSV infection in the THA study sample, the explanatory power of the atmospheric temperature was 0.49 (*p*<0.05) and that of the relative humidity was 0.42 (*p*<0.05). For RSV infection in the THG study sample, the explanatory power of the atmospheric temperature and relative humidity were 0.51 (*p*<0.05) for both variables. For RSV infection in the THG study sample, the explanatory power of the mean number of rainy days was 0.52 (*p*<0.05).

**Fig 5:**
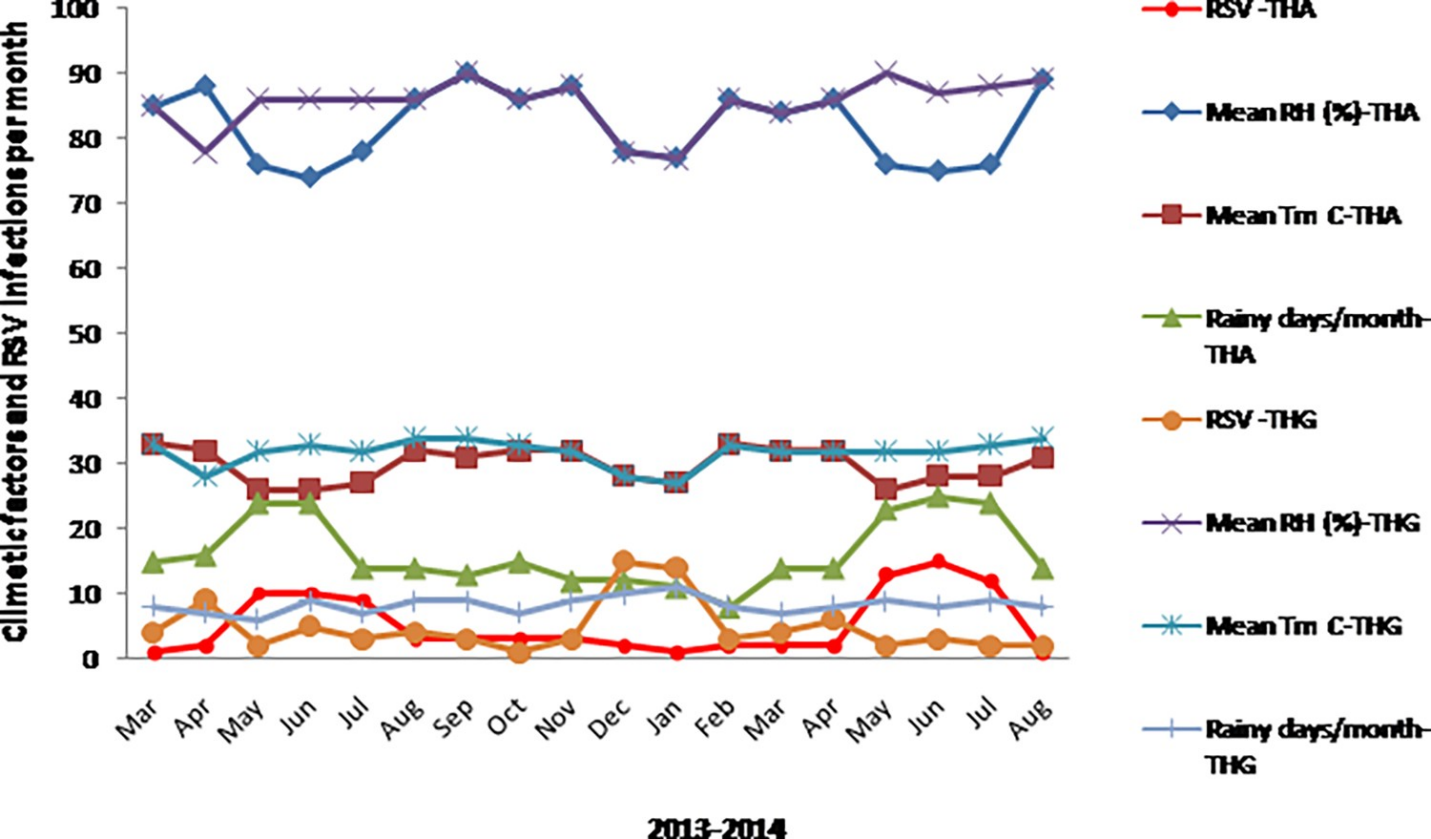
Pattern of RSV associated ARTI with climatic factors in THA and THG from March 2013 to August 2014. ARTI–Acute respiratory tract infection; THA–Teaching hospital, Anuradhapura; THG–Teaching hospital, Gampola; RSV–Respiratory syncytial virus; PIV-1, 2 & 3 –Parainfluenza virus 1, 2 & 3; Ad V–Adenovirus; Inf-A–Influenza A; Inf-B–Influenza B; hMPV–Human metapneumovirus. Climatic factors analyzed–mean monthly relative humidity (%), mean monthly temperature, mean monthly number of rainy days.

Spearman’s rank correlation and multiple linear regressions showed a direct correlation of monthly RSV cases with number of rainy days in the THG study sample and an inverse correlation with mean atmospheric temperature in both study samples. Additionally, regression analysis identified a significant inverse correlation of RSV cases with relative humidity in THA and THG study samples. Based on the multiple regression analysis, a monthly increase of one rainy day was associated with a 0.58 increase in monthly RSV cases in the THG. However, in THA and THG, an increase of 1% of relative humidity and 1°C of temperature were associated with 1.21 and 1.03 and 1.92 and 1.86 decreases in RSV cases, respectively. In the THA study sample, a total of 20.3% of explained variance (R^2^ = 0.203) in the number of monthly RSV cases was attributed to number of rainy days in a month, relative humidity, and temperature. In the THG study sample, a total of 23.3% of explained variance (R^2^ = 0.233) in the number of monthly RSV cases was attributed to the same. Other climatic factors like the wind speed and wind direction and atmospheric pressure were not significantly associated with RSV infections. The pattern of distribution of RSV associated ARTI with climatic factors (mean monthly relative humidity (%), mean monthly temperature, mean monthly number of rainy days) in THA and THG study samples are shown in Fig 5 and Supplementary 4 in S1 File.

The supplementary 7 in S1 File shows the interactions of the paired climatic factors on viral ARTIs and RSV associated ARTI at THA and THG. The dominant interaction is in bold letters where the q value is higher than the sum of each individual factor. The dominant interactors on overall ARTI (*q* = 0.71) at THA (*q* = 0.58) and THG (*q* = 0.72), viral ARTI and RSV associated ARTI at THA (*q* = 0.72) was atmospheric temperature and atmospheric pressure. The dominant interactor on RSV associated ARTI at THG was the mean number of rainy days and the mean atmospheric pressure (*q* = 0.74) (Supplementary 7 in S1 File).

### Risk factors for RSV associated ARTI

Viral co-infection was not included in risk factor analysis (Table 2). Male sex was a significant risk factor for acquiring RSV infection (Odds ratio = 2.4; *p =* 0.03) and other 7 respiratory viral infections (Odds ratio = 2.3; *p =* 0.03). Malnutrition was a significant risk factor for acquiring PIV-2 compared to RSV infection in THA (Odds ratio = 1.8; *p =* 0.02) and THG (Odds ratio = 2.1; *p =* 0.02). Birth weight <2500 g was a significant risk factor for acquiring AdV infection compared to RSV in children with ARTI in THA (Odds ratio = 1.7; *p =* 0.04) and THG (Odds ratio = 1.8; *p =* 0.04). Living in crowded conditions was a significant risk factor for acquiring AdV infection compared to RSV in children from THA (Odds ratio = 2.8; *p =* 0.03) and THG (Odds ratio = 3.2; *p =* 0.03). Birth through LSCS was a significant risk factor for acquiring hMPV infection compared to RSV in children from THA (Odds ratio = 1.9; *p =* 0.05) and THG (Odds ratio = 2.4; *p =* 0.05). Considering the co-morbidities, having congenital heart disease was a significant risk factor for acquiring RSV and AdV infection compared to 6 other respiratory viral infections in THA and THG. Having asthma was a significant risk factor for acquiring PIV-2 and hMPV infections compared to RSV infection in THA and THG.

**Table 2:** Risk factors for acquiring RSV and other viruses in children 1-≤ 60 months with ARTIs.

	RSV		PIV-1		PIV-2		PIV-3		AdV		Inf-A		Inf-B		hMPV	
**No. of virus positive ARTI**	94	85	6	11	12	16	8	8	17	17	8	11	5	8	15	9
**No. of co-infections** ^**a**^	13	13	3	3	3	4	0	0	4	4	1	1	0	1	4	4
**Study site**	THG	THA	THG	THA	THG	THA	THG	THA	THG	THA	THG	THA	THG	THA	THG	THA
**Risk factors**																
**Malnutrition (weight-for-age *z*-score –2)^b^**	-	-	-	-	2.1 (0.02)	1.8 (0.02)	-	-	-	-	-	-	-	-	-	-
**Male sex**	2.4 (0.03)	2.3 (0.03)	-	-	-	-	-	-	-	-	-	-	-	-	-	-
**Low birth weight^c^ (2500 g)**	-	-	-	-	-	-	-	-	1.8 (0.04)	1.7 (0.04)	-	-	-	-	-	-
**Mode of delivery–LSCS^d^**	-	-	-	-	-	-	-	-	-	-	-	-	-	-	2.4 (0.05)	1.9 (0.05)
**Outdoor air pollution^e^**	-	3.2 (0.04)	-	-	-	2.4 (0.03)	-	-	-	-	-	-	-	-	-	-
**Indoor air pollution**	-	-	-	-	-	-	1.8 (0.04)	1.5 (0.04)	-	-	-	-	-	-	-	-
**Passive smoking**																
**Breastfeeding (during the first 4 months)**	-	-	-	-	-	-	-	-	-	-	-	-	-	-	-	-
**Lack of measles immunization (within the first 12 months)**	-	-	-	-	-	-	-	-	-	-	-	-	-	-	-	-
**Crowded living**	-	-	-	-	-	-	-	-	3.2 (0.03)	2.8 (0.03)	-	-	-	-	-	-
**Concurrent/ Congenital heart diseases**	3.4 (0.02)	3.2 (0.02)	-	-	2.4 (0.03)	2.3 (0.03)	-	-	-	-	-	-	-	-	-	-
**Asthma**	-	-	1.4 (0.04)	1.4 (0.04)	-	-	-	-	-	-	-	-	-	-	2.1 (0.03)	2.1 (0.03)
**Immunodeficiency**	-	-	-	-	-	-	-	-	-	-	-	-	-	-	-	-
**Epilepsy**	-	-	-	-	-	-	-	-	-	-	-	-	-	-	-	-
**Mother’s experience as a caregiver**	-	-	-	-	-	-	-	-	-	-	-	-	-	-	-	-
**Mother’s education**	2.3 (0.04)	2.1 (0.04)	-	-	-	-	-	-	2.4 (0.03)	2.2 (0.03)	-	-	-	-	-	-
**Day-care attendance**	-	-	1.3 (0.03)	1.3 (0.03)	1.4 (0.03)	1.4 (0.03)	-	-	1.5 (0.03)	1.5 (0.03)	-	-	-	-	-	-
**> Mean monthly RH (%)**	2.5 (0.03)	2.1 (0.03)	-	-	-	-	-	-	-	-	-	-	-	-	-	-
**> Mean Tm (°C)**	3.1 (0.02)	2.9 (0.04)														
**Mean monthly rainy days> 15/month**	3.2 (0.03)	-	-	-	-	-	-	-	-	-	-	-	-	-	-	-
**Trisomy 21**	-	-	-	-	-	-	-	-	-	-	-	-	-	-	-	-
**Birth order > 3**	-	-	-	-	-	-	-	-	-	-	-	-	-	-	-	-

^a^Viral co-infections were excluded from the analysis. Only significant odds ratio and P value are given. P value was calculated in multivariate analysis and P< 0.05 was considered significant and given in parenthesis in relevant columns. THA–Teaching hospital, Anuradhapura; THG–Teaching hospital, Gampola; RSV–Respiratory syncytial virus; PIV-1, 2 & 3 –Parainfluenza virus 1, 2 & 3; Ad V–Adenovirus; Inf-A–Influenza A; Inf-B–Influenza B; hMPV–Human metapneumovirus; LSCS–Lower segment caesarian section.

Considering the socio-demographic factors, poor educational level of the mother (up to primary level only) was a significant risk factor for acquiring RSV and hMPV infections compared to 6 other viruses in THA and THG. Day care attendance was a significant risk factor for acquiring PIV-1 or PIV-2 or AV infection compared to RSV in THA and THG. In addition, outdoor air pollution was a significant risk factor for acquiring RSV and PIV-2 infection in THA compared to 6 other viruses. Indoor air pollution was a significant risk factor for acquiring PIV-3 infection compared to RSV in both THA and THG.

Considering the climatic factors, high relative humidity (not the mean monthly relative humidity) was a significant risk factor for acquiring RSV infection in children compared to all seven viruses in THA and THG. Similarly, high atmospheric temperature was a significant risk factor for acquiring RSV infection. Furthermore, having more than 15 rainy days in a month was a significant risk factor for acquiring RSV infection. Empirical antibiotic use was noted in all children with ARTI, in the study sample.

### Burden of viral ARTI in childhood

**Fig 6:**
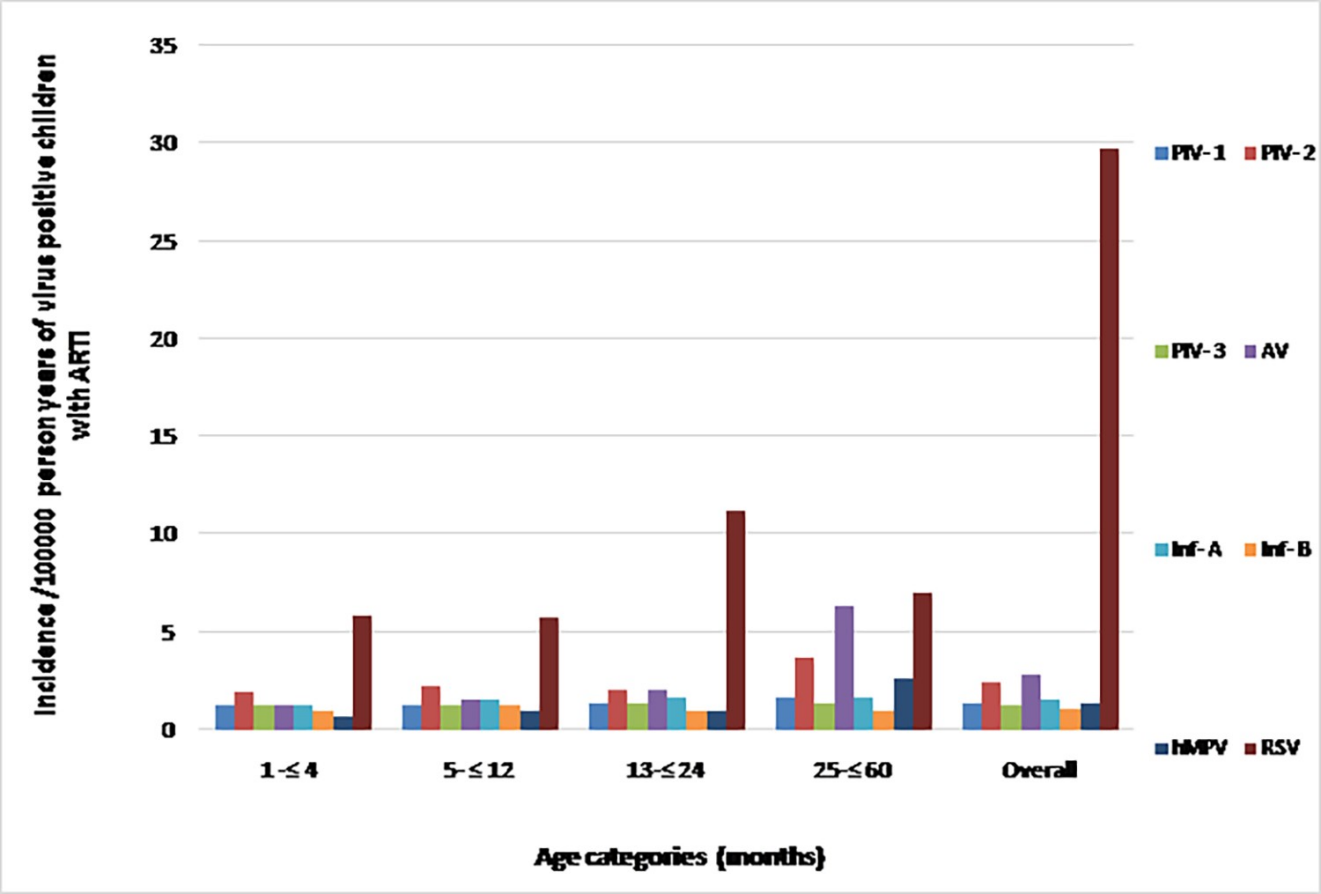
Incidence/100000-person years for RSV, PIV-1, PIV-2 and PIV-3, AV, Inf-A, Inf-B, and hMPV infections for different age groups of children with ARTI compared to overall incidence of these viruses despite the age categories. ARTI–Acute respiratory tract infection; RSV–Respiratory syncytial virus; PIV-1, 2 & 3 –Parainfluenza virus 1, 2 & 3; Ad V–Adenovirus; Inf-A–Influenza A; Inf-B–Influenza B; hMPV–Human metapneumovirus.

Viral incidence/100,000 person years for ARTI are given in Table 3 and Fig 6. Overall, RSV associated ARTI incidence was 29.76/100,000 person years and it was significantly higher for RSV than the rest of the viral ARTI (*p* = 0.001).

**Table 3:** Incidence/100000-person years for RSV, PIV-1, PIV-2 and PIV-3, AV, Inf-A, Inf-B, and hMPV infections for different age groups of children with ARTI compared to overall incidence of these viruses despite the age categories.

Age in months	Incidence/100000-person years for respiratory viruses tested								*p* value
	RSV	PIV-1	PIV-2	PIV-3	AV	Inf-A	Inf-B	hMPV	
**1 -≤ 4**	5.83	1.26	1.90	1.26	1.26	1.26	0.95	0.63	*0.03
**5- ≤ 12**	5.73	1.27	2.23	1.27	1.59	1.59	1.27	0.95	*0.03
**13-≤ 24**	11.20	1.35	2.02	1.35	2.02	1.69	1.01	1.01	*0.02
**25-≤ 60**	7.00	1.66	3.66	1.33	6.33	1.66	0.99	2.66	*0.02
**All age groups**	29.76	1.39	2.45	1.30	2.80	1.55	1.06	1.31	*0.001

*p* value 0.05 was considered significant. RSV–Respiratory syncytial virus; PIV-1, 2 & 3 –Parainfluenza virus 1, 2 & 3; Ad V–Adenovirus; Inf-A–Influenza A; Inf-B–Influenza B; hMPV–Human metapneumovirus.

In the THA study sample, 6 deaths were recorded for viral ARTI. Two of these children had RSV infection alone, 2 had RSV and hMPV co-infection (Supplementary 3 in S1 File) and 2 had RSV and PIV-1 co-infection (Table 4). In the THG study sample, 3 deaths were recorded for viral ARTI during the study period. Two of these children had RSV infection alone and one had RSV and PIV-1 co-infection. Case fatality rate for childhood ARTI at THA and THG was 1.43% (6/418) and 0.67% (3/443), respectively. Overall RSV associated case fatality rate in THA and THG study samples was 3.63% (6/165) and 1.81% (3/165), respectively. Case fatality rate for RSV mono-infection for both centers was 1.21% (2/165). DALYs in THA and THG were 429.77 and 242.06, respectively. DALYs for RSV infection were 322.33 and 161.37 for THA and THG, respectively.

**Table 4:** Mortality and morbidity data in children with viral ARTIs (lower ARTI).

Data on mortality and morbidity	THA	THG
Number of hospitalized children with ARTI	418	443
Number of viral positive ARTI	165	165
Number of deaths	6**	3*
Etiology for mortality	RSV = 2, RSV & PIV-1 = 2 and RSV & hMPV = 2	RSV = 2, RSV & PIV-1 = 1
DALYs	429.77	242.06

THA–Teaching hospital, Anuradhapura; THG–Teaching hospital, Gampola; ARTI–Acute respiratory tract infection; DALYs–Disability adjusted life years; RSV–Respiratory syncytial virus; PIV-1, 2 & 3 –Parainfluenza virus 1, 2 & 3; Ad V–Adenovirus; Inf-A–Influenza A; Inf-B–Influenza B; hMPV–Human metapneumovirus.

Overall, period prevalence of ARTI in the total study population was 21.5% while period prevalence of viral ARTI was 8.25%. Period prevalence of RSV associated ARTI in THA was 4.25% and in THG was 4.7%. Overall, ARTI incidence in the total study population was 287/100,000 person years for children ≤5 years. In THA, RSV associated ARTI incidence was 28.3/100,000 person years for children ≤5 years. In THG, RSV associated ARTI incidence was 31.3/100,000 person years for children ≤ 5 years. Age related incidence of ARTI in infants <12 months of age in THA and THG was 9.6/100000 and 9.3/100000 person years, respectively. Hospitalization following RSV infection in children aged <5 years in the rural population was significantly (*p* = 0.03) higher than that in the urban population. The risk of viral ARTI associated case fatality for THA and THG was 3.63% (6/165) and 1.81% (3/165 (Table 4). All deaths were associated with RSV associated pneumonia or bronchiolitis in mono-infection or co-infection with other respiratory viruses. Viral ARTI associated case fatality rate was 2.4% (4/165) and 1.2% (2/165), respectively for THA and THG in the presence of congenital cardiac disease.

## Discussion

As with most parts of the world, ARTIs in children under 5 years is one of the most common causes of morbidity in Sri Lanka [31]. The distribution of respiratory viruses in Sri Lanka appears to be like those in developing countries and other tropical regions [32]. The age distribution and the clinical picture of respiratory viral infections were also like those previously described in tropical countries [33]. As noted in the other regions [32, 33], the present study demonstrated an annual pattern for respiratory viral infections in Sri Lanka.

Information on the causes of respiratory illness in tropical countries is limited compared to temperate countries. The available data indicates that about one third of the respiratory tract infections are caused by viruses based on antigen detection using IFA [34, 35]. The present study identified viruses in 37.1% ± 0.75 and 36.4% ± 0.12 of children hospitalized for ARTI in THA and THG, respectively. Compared to previous studies, antigen detection by IFA in our study shows a higher detection rate [36–39], and it may be due to higher detection indices of the current assay compared to those used in the previous studies.

In the present study, RSV was the most predominant virus detected in both centers with an incidence of 29.8/100,000 person years. This RSV incidence of the present study is lower than that estimated in children based on hospital-surveillance done in Thailand (46/100,000 person-years in children) [40] and Kenya (293/100 000 person-years in children aged <5 years) [41]. In the USA and UK, it was 300 per 100,000 person-years in children aged<5 years [42, 43]. According to the current study, RSV associated ARTI incidence in Sri Lanka is somewhat like Thailand and it might be due to low prevalence of RSV infection in the Asian region compared to UK, USA and Kenya. Another reason for low prevalence of RSV infection in Sri Lanka might be due to low level of hospitalization of children following ARTIs as urban as well as rural areas of Sri Lanka has general practitioners. Conversely, with high degree of parental concern towards childhood illnesses, parents seek medical advice early in the illness.

RSV predominance in Sri Lankan children agrees with the fact that this virus is the single most frequent respiratory pathogen detected in infants and young children worldwide. When considering the clinical characteristics of RSV associated ARTI, fever was less frequently noted in the present study. Rhonchi, rhinorrhea, chest recession and tachypnea were observed commonly. Overall, these signs and symptoms are related to the any known pathogen causing ARTIs and not characteristically confined to a specific virus as noted by many other studies as well [43–45].

Disease spectrum in RSV associated ARTIs ranged from common cold to life threatening severe bronchiolitis and pneumonia, suggesting the role of RSV in most of the cases with exacerbation of bronchiolitis [46, 47]. Moreover, the disease spectrum noted by the current study agrees with that noted in many studies done elsewhere [7, 48, 49]. RSV is associated with many viral co-infections and this finding is also in agreement with previous studies [50–52]. The association between the occurrence of co-infection and development of severe disease is unclear [53], and the present study also noted morbidity and mortality in children with viral co-infections with RSV.

Overall, the present study demonstrated an annual seasonality of viral ARTI in Sri Lanka in two different climatic zones especially for RSV infections. There is a marked seasonal variation in viral incidence between the temperate and tropical countries [54]. In tropical countries, the seasonality varies according to the temperature-dependent local weather pattern such as humidity or/and rainfall [17]. Epidemiological studies from tropical regions indicate that factors such as rainy seasons [55] or low air humidity [56] and temperature may influence outbreaks of viral ARTIs. Global warming has a profound influence in the earth’s climate and changes in the climate has an impact on the seasonality and burden of respiratory viral infections. In addition to climatic factors, population density and degree of exposure to risk factors predisposing to the acquisition of ARTI in childhood also have a role in the overall burden of viral ARTI [57–59].

RSV infection was detected throughout the year in the current study. The seasonality correlated with the rainy season in both wet (South-west monsoon from May to September) and dry zone (North-east monsoon from December to March) and this explains the positive correlation with number of rainy days per month instead of monthly rain fall, which has a less bearing on the viral incidence [57, 60]. A slightly similar RSV seasonality from May to July has been reported by other studies conducted in the southern part of Sri Lanka, which experiences almost similar climatic conditions to the wet zone study center of the current study [61, 62]. The peak occurrence of RSV infections in the rainy season might be due to indoor overcrowding. At THG, the dominant interaction was detected between mean number of rainy days and atmospheric pressure (*q* = 0.74). At THA, the dominant interaction was detected between the mean atmospheric temperature and atmospheric pressure (*q* = 0.58). Moreover, in a study conducted in China [28], the dominant interaction was observed between the mean wind speed and the mean atmospheric pressure (*q =* 0·836). According to that study, a low atmospheric pressure would enhance the spread of airborne viruses. Our findings and the findings of the Xu et al. [28] suggest that these interactions might differ in different geographical areas and that the interpretations would differ accordingly.

There have been a few studies on PIV infections in developing countries and most of these studies do not differentiate the subtypes of the virus. Our study recorded a 3.1% period prevalence of PIV infections with the predominance of PIV-3. Similar hospital-based studies have been reported in other developing countries [63, 64]. PIV infections have been strongly associated with croup based on the findings of some previous studies [65, 66]. However, the present study recorded a smaller number of cases with croup among PIV infected patients. In the THA study sample, an absence of a well demarcated seasonality was seen for PIV associated ARTI. In the THG study sample, PIV associated ARTI was scattered in the second quarter of the year. This distribution cannot be explained using climatic or other risk factors. Furthermore, the number of PIV cases was also less in the present study and thus extracting a clear association is difficult.

AdV was the third highest prevalent virus causing ARTI in the study sample. The period prevalence for AdV associated ARTI was 5.66% and this was similar in both study centers. In the tropics, AdV has been reported to be responsible for 5–10% of the ARTI affecting infants [65, 67–69]. In our study, AdV was evenly distributed in different age groups of children <5 years. Furthermore, diarrhea and conjunctivitis were significantly associated with AdV infection. However, AdV infections showed a year-round distribution in the wet zone including the rainy season with no seasonality.

Period prevalence for Inf-A was 3.66 and 2.66 and Inf-B was 2.66 and 1.66 in THA and THG, respectively. Inf-V infections were associated with a smaller number of morbidity and mortality and this may be due to scattering of antigenically stable Inf-A and Inf-B strains during the inter-epidemic period. In 2015, an influenza epidemic was reported in Sri Lanka with significant mortality and morbidity, however, no obvious seasonality was noted with these outbreaks either [70], supporting the findings of the present study.

hMPV infection is more prevalent in countries with temperate climates. We detected children with hMPV associated ARTI in both study centers. This is one of the large-scale studies detecting hMPV infection in Sri Lanka. Co-infections with RSV and hMPV were also detected. The disease spectrum and the severity of hMPV infection are like that of RSV associated ARTI [71–73]. Although the number of cases identified with hMPV associated ARTI were less, hMPV associated ARTI peak overlapped the RSV peak and thus the same climatic conditions might influence the distribution of RSV and hMPV.

Taken study findings together, identifying the pattern of distribution of viral ARTI in a location will help implementing the early preventive measures, which can be communicated to the public with awareness on RSV infection including the RSV dominant times in the forth coming year and in rainy seasons. The RSV burden is high and present throughout the year and the Inf-V related morbidity is less and confined only to the epidemic period with low prevalence during the inter-epidemic period, however, introducing influenza vaccination will help to prevent Inf-V associated ARTI in high risk children. Now no specific evidence based preventive measures are practiced in Sri Lanka to control childhood ARTI. The current data on RSV burden and the pattern of distribution will contribute to health promotion activities in Sri Lanka among parents, guardians and childcare providers.

Considering the viral burden, DALYs is less compared to other studies done in the tropics [74, 75]. DALYs is a measure of mortality and associated burden following viral ARTI. DALYs for ARTI in THA and THG were 429.77 and 242.056, respectively. DALYs for RSV infection was 322.33 and 161.37 for THA and THG, respectively. These DALYs values of the current study are significantly lower when compared to DALYs associated with ARTI in South-East Asian region [76]. According to the WHO, Sri Lanka lies in <25 deaths/1000 live births for under 5 years of age [77] and this reflects the low mortality rate and DALY associated with ARTIs in the island. On the other hand, it may also be due to high level of parental care, including seeking early medical advice for childhood illnesses leading to lesser chances for worsening of the illness. Although specific antiviral treatment is not available for many viral ARTI, symptomatic management eases the severity and the duration of the illness.

Malnutrition has generally been considered as a risk factor for viral ARTI in developing countries [78, 79], however, the increased mortality could occur especially when bacterial infections are involved during post-viral convalescence. In our study, malnutrition was identified as a potential risk factor for acquiring PIV-2 and AdV infections. In addition to tested nutrition indicators, the low level of Hb% reflected the level of malnutrition and this finding has been shown by many other studies as well [80]. All patients with anemia were screened for a complete blood evaluation (CBE) and found to have iron deficiency anemia. Viral infections further deprive the nutritional state by reducing the appetite in a child with ARTI, leading to increased risk for recurrent viral infections [81]. Viral testing on respiratory secretions like NPA is the accurate way of identifying the viral etiology and lack of viral diagnostics will raise the use of antimicrobials irrationally.

The present study also noted the empiric use of antibiotics in children with ARTI in accordance with the pediatric emergency treatment protocol for managing moderate to severe bronchiolitis. In these instances, antibiotic treatment does not contribute positively to clinical improvement as noted by previous studies [80, 81]. On the other hand, use of antibiotics in children with viral ARTI contributes to an enormous health cost in developing economies like Sri Lanka. Moreover, rational use of antibiotics will contribute to minimize the development of antimicrobial resistance. Health care policy makers must consider reviewing the clinical algorithm for patient management with virologists and clinicians considering the cost incurred by the irrational use of antibiotics for viral infections and this expenditure can be diverted to establish viral diagnostics. Future cross-sectional and longitudinal studies of viral ARTI in out-patients and communities will contribute to identify the time bound trends on the respiratory viral burden. This will alarm the clinicians about common, uncommon and newly emerging respiratory viruses. This study also shows the number of viral ARTI associated hospitalization in two climatic zones of Sri Lanka.

Overall, period prevalence of ARTI in both centers was 21.5% while the period prevalence of virus identified ARTI was 8.25%. Overall, ARTI incidence in the study sample was 287/100,000 person years for ≤ 5-year-old children. In addition to the morbidity, the management cost and the loss of parent/guardian manpower used for the childhood ARTI have economic consequences. This is the first large study to detect hMPV associated ARTI in Sri Lankan children. Our data shows the respiratory viral burden on childhood ARTI and this will help the health care providers and policy makers to re-evaluate the existing infection control and prevention strategies for viral ARTI. Awareness on local respiratory viral dynamics is important to implement early preventive measures like use of respiratory precautions and health education for different categories of care providers including parents. Prevention plays a significant role in reducing the burden of childhood viral ARTIs, however, adherence to preventive methods must be promoted by the health care providers and the policy makers.

Recruiting only the hospitalized children with SARI led to miss a fair number of patients who reported to the outpatient department with viral ARTIs. Moreover, Sri Lanka has a well spread network of general practitioners and parents seek paid medical care following ARTI and this would have also reduced hospitalizations leading to drop in the actual number of ARTI and this would under-estimate the true burden of viral ARTI. This study was confined to children between 1 month to 5 years and thus missed the pediatric ARTI cases from >5 to <12 years. Since detection sensitivity of IFA for viral antigen is low compared to molecular methods, the study would have missed some more true viral ARTI cases. We have also not tested for rhinoviruses and enteroviruses which contribute to >10% of all viral ARTIs. In addition to detecting hMPV using IFA, only 100 RSV positive and 100 virus negative NPA were further tested using PCR for hMPV, hBoV, and CoV.

## Conclusions

Overall, 38.33% of the hospitalized children had virus identified ARTI based on antigen detection by IFA. The level of viral detection was similar in both study samples representing dry and wet zones of Sri Lanka. RSV was the commonly detected respiratory virus with an annual pattern of distribution and RSV associated ARTI was distributed during the rainy seasons. In the wet zone, RSV associated ARTI was seen around May to July along with the South-West monsoon. In the dry zone, RSV associated ARTI was seen around December to January along with the North-East monsoon. Furthermore, monthly RSV cases negatively correlated with mean monthly atmospheric temperature and mean monthly relative humidity, and positively correlated with the number of rainy days present in a month instead of the mean rain fall. Other respiratory viruses tested in the study have not shown such a correlation. Moreover, this study has demonstrated that respiratory viruses are associated with a considerable number of hospitalizations in Sri Lanka. Future cross-sectional and longitudinal studies of viral-ARTI including out-patients will contribute to assess the true burden and the time bound diversity of viral ARTI in Sri Lanka.

## Supporting information

S1 File (PDF)
