# Supplementary material for: Viral burden and diversity in acute respiratory tract infections in hospitalized children in wet and dry zones of Sri Lanka
Source: PLoS One. 2021 Dec 17;16(12):e0259443. doi: 10.1371/journal.pone.0259443 (PMC8682885; doi:10.1371/journal.pone.0259443)
Supplement: S1 File — (PDF) [file pone.0259443.s001.pdf]

Supporting Information

Viral Burden and Diversity in Acute Respiratory Tract Infections in Hospitalized Children in Wet and Dry Zones of Sri Lanka

JAAS Jayaweera[1,2¶], AJ Morel[3], AMSB Abeykoon[2], FNN Pitchai[2&], HS Kothalawela[2&], JSM Peiris[4]and F Noordeen[2*¶]

[1]Department of Microbiology, Faculty of Medicine and Allied Sciences,

Rajarata University of Sri Lanka, Saliyapura, Sri Lanka

[2]Department of Microbiology, Faculty of Medicine, University of Peradeniya, Peradeniya, Sri Lanka

[3]Teaching Hospital, Gampola, Gampola, Sri Lanka

[4]School of Public Health, University of Hong Kong, Hong Kong

[*]Corresponding Author

E mail: faseeha.noordeen12@gmail.com; faseehan@pdn.ac.lk (FN)

[¶]These authors contributed equally to this work.

[&]These authors also contributed equally to this work.

Key words: Childhood ARTI, viral burden, seasonality, risk factors

Supplementary 1. Comparison of demographic characteristics of childhood ARTI in THA and THG study samples.

	**THA**	**THG**	**P value P<0.05**
Age at presentation<br>Mean ± SD (months)	14.26 ± 12.01	12.82± 12.62	0.12
Male: Female	1.73<br>265 : 153	1.66<br>277 : 166	0.14
Mean birth weight (Kg ± SD)	2.85 ±0.45	2.9 ± 0.4	0.15
Mean body weight (Kg ± SD)	8 ± 3	9 ± 4	0.23
Ethnicity<br>Sinhala<br>Tamil<br>Muslim	 370<br>35<br>13	 275<br>90<br>78	 0.12<br>0.03*<br>0.02*
Residence<br>Rural<br>Semi Urban<br>Urban	 324<br>60<br>34	 265<br>120<br>58	 0.12<br>0.04*<br>0.06<br>0.21

ARTI – Acute respiratory tract infection; THA – Teaching Hospital, Anuradhapura;

THG – Teaching Hospital, Gampola; **P*<0.05 was considered as significant.

Supplementary 2. PIV associated ARTI in children : clinical diagnosis, mean hospital stay and use of antibiotics.

	PIV-1		PIV-2		PIV-3	
**Clinical diagnosis**	**(n=11) THA**	**(n=6) THG**	**(n=16) THA**	**(n=12) THG**	**(n=8) THA**	**(n=8) THG**
Common cold	2	1	2	1	1	0
Acute sinusitis	1	1	0	1	1	1
Acute pharyngitis	0	0	1	1	1	1
Acute tonsillitis	0	0	1	0	0	0
Acute laryngotracheitis	0	0	2	2	0	0
Un specified AURTI	0	0	1	1	1	1
Acute bronchiolitis	1	1	4	2	1	2
Mild	1	1	1	0	0	1
Moderate	0	0	2	1	0	1
Severe	1	0	1	1	1	0
Acute bronchitis	0	0	0	0	0	0
Pneumonia						
Lobar	1	1	2	0	0	1
Broncho	1	1	1	1	1	1
Un specified ALRTI	1	0	0	0	0	0
Exacerbation of asthma	0	0	1	0	1	0
Mean hospital stay in days ± SD	3±2.6	3±2.1	3±2.2	3±2.1	3±2.4	3±2.3
Treatment with antibiotics	7 (63.3%)	4 (66 %)	9 (56.2%)	10 (83.3%)	3 (37.5%)	6 (75%)

ARTI – Acute respiratory tract infection; AURTI – Acute upper respiratory tract infection; ALRTI - Acute lower respiratory tract infection; PIV – Parainfluenza virus; THA – Teaching Hospital, Anuradhapura; Teaching Hospital, Gampola.

Supplementary 3. Disease spectrum and severity of ARTI following hMPV and RSV / hMPV co-infection.

		THA		THG	
	**hMPV**	**RSV/hMPV**	**hMPV**	**RSV/hMPV**	**Comments**
	(n=9)	(n=6)	(n=16)	(n=4)	
**Clinical diagnosis**					
Common cold	2	1	2	1	
Bronchiolitis	3	1	3	1	
Severe	2	1	2	1	
Moderate	0	0	0	0	
Mild	1	0	1	0	
Pneumonia	3	3	3	3	
Lobar pneumonia	1	1	1	1	
Broncho pneumonia	1	1	1	1	
Lobar pneumonia and severe bronchiolitis	1	1	1	1	Both were treated in ICU
IE BA	1	1	1	1	

ARTI – Acute respiratory tract infection; RSV – Respiratory syncytial virus; hMPV – human metapneumovirus; THA – Teaching Hospital, Anuradhapura; Teaching Hospital; Gampola; IEBA - Infective exacerbation of bronchial asthma; *ICU - Intensive care unit.

Supplementary 4. Correlation of climatic factors with RSV associated ARTI in THA and THG study samples.

**Climatic factors**	**Mean ± SD**		**Spearman's correlation coefficient**		***P* value**		**Multiple linear correlation coefficient**		***P* value**	
	**THA**	**THG**	**THA**	**THG**	**THA**	**THG**	**THA**	**THG**	**THA**	**THG**
Atmospheric temperature °C	29.5 ± 4.6	25.5 ± 3.6	-0.14	-0.12	0.032	0.031	-1.92	-1.86	<0.001	<0.001
Relative humidity (%)	86 ± 6.8	78 ± 8.8	-0.15	-0.08	0.077	0.067	-1.21	-1.03	<0.001	<0.001
Rain days (n)	9 ± 2.8	19 ± 3.5	0.12	0.14	0.064	0.045	0.43	0.58	0.08	<0.001
Rain fall (mm)	1200± 290	2600 ± 200	0.95	0.10	0.062	0.065		NS		

THA – Teaching Hospital, Anuradhapura; Teaching Hospital, Gampola.

Supplementary 5. Explanatory power of climatic factors on overall viral ARTI and RSV associated ARTI at THA and THG study samples.

**Category**	**Atmospheric temperature *q* (*p*)**	**Relative humidity *q* (*p*)**	**Mean number of rainy days *q* (*p*)**	**Wind speed *q* (*p*)**	**Wind direction *q* (*p*)**	**Atmospheri c pressure *q* (*p*)**
Overall viral ARTI cases	*0.41 (0.042)	*0.42 (0.035)	*0.46 (0.024)	0.18 (0.142)	0.17 (0.078)	0.13 (0.102)
Overall viral ARTI cases at THA	*0.39 (0.031)	*0.44 (0.011)	*0.46 (0.021)	0.12 (0.131)	0.19 (0.231)	0.13 (0.091)
RSV associated ARTI cases at THA	*0.49 (0.025)	*0.42 (0.003)	0.18 (0.131)	0.09 (0.079)	0.13 (0.074)	0.19 (0.055)
Overall viral ARTI cases at THG	*0.44 (0.041)	*0.41 (0.023)	*0.48 (0.031)	0.23 (0.141)	0.15 (0.091)	0.14 (0.092)
RSV associated ARTI cases at THG	*0.51 (0.011)	*0.51 (0.009)	*0.52 (0.012)	0.11 (0.111)	0.21 (0.071)	0.11 (0.121)

ARTI - Acute respiratory tract infection; THA - Teaching Hospital; Anuradhapura; THG - Teaching Hospital, Gampola; RSV - Respiratory syncytial virus; *$P<0.05$ statistically significant.

Supplementary 6. Explanatory power of climatic factors on viral ARTI in different age groups and gender at THA and THG study samples.

Category	Age (months)	Gender	Atmospheric temperature ***q*** (***p***)	Relative humidity ***q*** (***p***)	Mean number of rainy days ***q*** (***p***)
Overall viral ARTI	1-≤12	Male	*0.39 (0.012)	*0.41 (0.021)	*0.48 (0.011)
		Female	0.28 (0.112)	0.29 (0.072)	0.31 (0.084)
	12-24	Male	*0.46 (0.011)	*0.48 (0.001)	*0.51 (0.001)
		Female	*0.39 (0.037)	0.29 (0.077)	0.31 (0.087)
	≥ 24-≤ 60	Male	*0.38 (0.032)	*0.42 (0.023)	*0.41 (0.019)
		Female	0.29 (0.124)	0.28 (0.084)	0.29 (0.093)
Overall viral ARTI at THA	1-≤12	Male	*0.41 (0.002)	*0.44(0.023)	*0.48(0.001)
		Female	0.26 (0.122)	0.29 (0.061)	0.30 (0.092)
	12-24	Male	*0.48 (0.001)	*0.51 (0.001)	*0.50 (0.001)
		Female	0.39 (0.033)	0.39 (0.064)	0.33 (0.067)
	≥ 24-≤ 60	Male	0.36 (0.121)	*0.41 (0.051)	*0.45 (0.021)
		Female	0.28 (0.113)	0.29 (0.062)	0.31 (0.092)
Overall viral ARTI at THG	1-≤12	Male	0.34 (0.052)	*0.44 (0.011)	*0.49 (0.001)
		Female	0.28 (0.142)	0.28 (0.072)	0.31 (0.084)
	12-24	Male	*0.46 (0.001)	*0.48 (0.011)	*0.52 (0.001)
		Female	0.39 (0.022)	0.29 (0.067)	0.31 (0.093)
	≥ 24-≤ 60	Male	*0.39 (0.021)	*0.43 (0.011)	*0.41 (0.031)
		Female	0.29 (0.131)	0.28 (0.084)	0.27 (0.103)

ARTI - Acute respiratory tract infections; THA - Teaching Hospital, Anuradhapura; THG - Teaching Hospital, Gampola; *P<0.05 statistically significant.

Supplementary 7. Interactive effects of paired climatic factors on overall ARTI, viral ARTI at THA and THG and RSV associated ARTI at THA and THG.

Category	Climatic factors	AT	RH	RD	WS	WD	AP
Overall ARTI	AT	0.41	0.54	0.57	0.37	0.23	*0.71
	RH	0.54	0.42	0.54	0.34	0.25	0.45
	RD	0.57	0.54	0.46	0.33	041	0.38
	WS	0.37	0.34	0.33	0.18	0.14	0.23
	WD	0.23	0.25	0.41	0.14	0.17	0.21
	AP	0.71	0.45	0.38	0.23	0.21	0.13
Overall viral ARTI at THA	AT	0.39	0.46	0.34	0.21	0.32	*0.58
	RH	0.46	0.44	0.24	0.34	0.44	0.31
	RD	0.34	0.24	0.46	0.21	0.23	0.25
	WS	0.21	0.34	0.21	0.12	0.17	0.12
	WD	0.32	0.44	0.23	0.17	0.19	0.21
	AP	0.58	0.31	0.25	0.12	0.21	0.13
RSV associated ARTI at THA	AT	0.49	0.44	0.32	0.24	0.31	*0.72
	RH	0.44	0.42	0.24	0.34	0.41	0.31
	RD	0.32	0.24	0.18	0.20	0.21	0.22
	WS	0.24	0.34	0.20	0.09	0.17	0.13
	WD	0.31	0.41	0.21	0.17	0.13	0.21
	AP	0.72	0.31	0.22	0.13	0.21	0.19
Overall viral ARTI at THG	AT	0.44	0.42	0.33	0.23	0.31	*0.60
	RH	0.42	0.41	0.24	0.34	0.41	0.33
	RD	0.33	0.24	0.48	0.21	0.21	0.24
	WS	0.23	0.34	0.21	0.23	0.17	0.13
	WD	0.31	0.41	0.21	0.17	0.15	0.22
	AP	0.60	0.33	0.24	0.13	0.22	0.14
RSV associated ARTI at THG	AT	0.51	0.43	0.34	0.25	0.32	0.48
	RH	0.43	0.51	0.24	0.35	0.41	0.33
	RD	0.34	0.24	0.52	0.21	0.21	*0.74
	WS	0.25	0.35	0.21	0.11	0.16	0.13
	WD	0.32	0.41	0.21	0.16	0.21	0.24
	AP	0.48	0.33	0.74	0.13	0.24	0.11

ARTI - Acute respiratory tract infections; THA - Teaching Hospital Anuradhapura; THG - Teaching Hospital Gampola; RSV - Respiratory syncytial virus; AT- Atmospheric temperature; RH - Relative humidity; RD - Mean number of rainy days; WS - Wind speed; WD - wind direction; AP - Atmospheric pressure.
*When the individual effect of a climatic factor was greater than the sum of the individual effects of the pair, the interaction was taken as a dominant interaction.
